# Severe cholestasis in neonatal extracorporeal membrane oxygenation

**DOI:** 10.1051/ject/2025031

**Published:** 2025-12-17

**Authors:** Michelle Jancaric, Benjamin Langworthy, Sixto Guiang, Bradley Segura, Rachel Wallace, Catherine Larson-Nath, Katherine M. Satrom

**Affiliations:** 1 University of Minnesota, Department of Pediatrics Minneapolis MN USA; 2 University of Minnesota, School of Public Health, Division of Biostatistics & Health Data Science Minneapolis MN USA; 3 University of Minnesota, Department of Surgery Minneapolis MN USA; 4 M Health Fairview, University of Minnesota Medical Center Minneapolis MN USA

**Keywords:** Neonate, Extracorporeal Membrane Oxygenation (ECMO), Bilirubin, Cholestasis, Hemolysis

## Abstract

*Introduction*: Cholestasis is a common complication of Extracorporeal Membrane Oxygenation (ECMO) secondary to patient physiology and circuit-induced factors. In our institution’s Neonatal Intensive Care Unit (NICU), we noted cases of severe cholestasis, with peak conjugated bilirubin levels much higher than previously reported in the literature. The objective of our study was to identify the contributing factors to the development of severe cholestasis in neonatal ECMO cases. *Methods*: Using our institutional ECMO database, all neonates who received ECMO at our institution were identified. A retrospective chart review was completed for a sample of 30 neonates. Univariate, multivariate, and logistic regression models were utilized. *Results*: Twenty percent of the patients in our study developed severe cholestasis (peak conjugated bilirubin > 10.0 mg/dL). Comparing the group of neonates that developed severe cholestasis to those who did not, we found that severe cholestasis was associated with the use of the Getinge Pediatric Quadrox-iD oxygenator. Mean plasma free hemoglobin levels were significantly higher in cases using pediatric oxygenators vs. adult (204.6 mg/dL vs. 110.4 mg/dL, *p* = 0.01). Longer ECMO courses and percent time within the ACT goal were also associated with severe cholestasis. *Conclusion*: Our study describes a cohort of neonatal ECMO cases complicated by severe cholestasis that was mediated by hemolysis due to circuit factors. In particular, circuit factors (the use of a pediatric oxygenator), longer duration of ECMO, and anticoagulation management were all significant factors. Future studies are needed to further elucidate the impact of these circuit factors and how they interplay with neonatal physiology.

## Introduction

Extracorporeal membrane oxygenation (ECMO) is an invasive method of cardiorespiratory support that is used to assist neonates who cannot adequately maintain gas exchange and/or perfusion despite maximal medical support. While ECMO offers life-sustaining therapy, it is not without significant risks, including infection, neurologic disease, kidney injury, hemostatic imbalance, and GI complications, notably cholestasis [1]. Cholestasis is an elevation in conjugated bilirubin levels leading to retention of biliary substances within the liver that are normally excreted through the intestine [2].

Previous studies have identified the incidence of cholestasis in neonates on ECMO between 14% and 39% [3–5]. The etiology of cholestasis is multifactorial, due to both physiologic and circuit-induced factors. Physiological factors in critically ill patients contributing to cholestasis include acidosis, ischemia, and liver injury [3]. Additionally, the ECMO circuit itself contributes to cholestasis through circuit-induced hemolysis. Circuit factors associated with increased risk of hemolysis include centrifugal pumps and smaller arterial and venous cannula sizes [6–10]. The proposed mechanism of hemolysis-induced cholestasis is the release of unconjugated bilirubin when red blood cells are hemolyzed, which is then converted to conjugated bilirubin in the liver. With high levels of hemolysis, the amount of bilirubin produced exceeds the liver’s ability to excrete it, leading to cholestasis.

Individuals with cholestasis have an increased risk of ECMO complications, including renal, metabolic, and infectious co-morbidities [4]. Furthermore, survival is significantly lower in patients with cholestasis, with previous studies demonstrating increased odds of death, even after controlling for etiology and disease acuity [3, 11–13]. However, the associated morbidity and mortality for cholestasis on ECMO is not well studied in the neonatal population. The few studies that have been published are limited by the age of the studies (ECMO indications and technology have rapidly evolved in recent years) and small patient populations at a single institution [3–5]. Subsequently, the majority of data regarding etiology and management of cholestasis is extrapolated from studies of pediatric and adult patients. Neonates differ significantly from these populations with regard to indication for ECMO. Furthermore, many of the components of the ECMO circuitry are smaller for neonates. Given these differences in neonatal ECMO, we sought to understand the etiology of severe conjugated hyperbilirubinemia in this population. To accomplish this, we investigated the impact of circuit factors and intrinsic patient characteristics on the development of severe cholestasis in neonatal ECMO cases.

## Methods

### Data collection

We used the institutional ECMO database to identify all individuals who received ECMO at the University of Minnesota Masonic Neonatal Intensive Care Unit (NICU) between January 2015 and June 2022. A retrospective chart review was completed using the electronic health record (EHR), and data were input into a secure database (REDCap electronic data capture tools hosted at the University of Minnesota). The Institutional Review Board at the University of Minnesota approved this study (STUDY00013910).

### Clinical management and ECMO circuit characteristics

Neonates were placed on ECMO in accordance with the indications presented in the Extracorporeal Life Support Organization (ELSO) guidelines [14]. All neonatal ECMO cases used the Medtronic Affinity centrifugal pump (Minneapolis, Minnesota) coated with Cortiva BioActive Surface, a heparin-coating technology. Two types of oxygenators were used during the study period, the Getinge Adult Quadrox-iD and the Getinge Pediatric Quadrox-iD, which were coated with Bioline, an albumin heparin technology (Rastatt, Germany). All cases before 2018 utilized the Getinge Adult Quadrox-iD, and all cases in 2018 or later utilized the Getinge Pediatric Quadrox-iD oxygenator due to an institutional change in equipment utilization. Medtronic Bio-Medicus cannulas were used. ECMO tubing was ¼ inch in diameter and was coated with Carmeda Bioactive surface (Upplands Vasby, Sweden). Circuits were primed using a standardized mixture of normal saline, albumin, sodium bicarbonate, fresh frozen plasma, and packed red blood cells. Prior to initiation of ECMO, a 100 unit/kg bolus of heparin was given, followed by a heparin infusion. Activated coagulation time (ACT) and unfractionated anti-factor Xa (anti-Xa) levels were used to titrate the heparin infusion, with goal ACT levels of 180–220 s and/or anti-Xa levels of 0.30–0.70 IU/mL. ECMO flow rate and circuit revolutions per minute (RPM) were titrated to maintain an SvO2 of 65–75%. For the duration of ECMO, neonates did not receive enteral feedings but rather were supported with total parental nutrition to meet all their nutritional needs.

### Statistical analysis

The neonates were divided into two groups based on the degree of cholestasis: the control group and the group with severe cholestasis. The majority of infants in our study had cholestasis based on the definition set by NASPGHAN, which defines cholestasis as a conjugated bilirubin > 1.0 mg/dL [2]. However, given that the focus of our study was to identify the etiology of the severe cholestasis cases, we chose to use a peak conjugated bilirubin level of 10 mg/dL or greater to define severe cholestasis.

We analyzed demographic, ECMO, and clinical variables on their relationship with severe cholestasis utilizing Fisher’s exact test for categorical variables and the Wilcoxon rank-sum test for continuous variables. In addition to our univariate analysis, we also utilized a multivariate model with peak conjugated bilirubin as the outcome. Given the relatively small sample size of 30, we performed model selection using the Akaike Information Criterion (AIC). The variables considered for inclusion were gestational age, birth weight, days on ECMO, underlying disease, oxygenator type, circuit change while on ECMO (yes/no), the presence of a visible clot, venous cannula size, arterial cannula size, percentage of time within ACT goal, average ECMO flow, and average RPM. The variables selected into the model using both forward and backward selection were days on ECMO, oxygenator type, venous cannula size, and percentage of time within the ACT goal.

In addition to a linear model with peak conjugated bilirubin as the outcome, we also fit a logistic model with Firth’s correction using severe cholestasis based on our study definition as the outcome and the same covariates as the linear model. This was done as a sensitivity analysis given the small sample size and extreme values for peak conjugated bilirubin. Additionally, we used two sample t-tests to assess the relationship between pediatric oxygenators and markers of hemolysis, including D-dimer, fibrinogen, and plasma free hemoglobin (PFH). Lastly, a mediation analysis was completed, controlling for the above variables and plasma-free hemoglobin. All analyses were completed using statistical analysis software R version 4.2.2.

## Results

Between January 2015 and June 2022, 30 neonates admitted to the University of Minnesota NICU were cannulated onto ECMO. The most common indication for ECMO was congenital diaphragmatic hernia, followed by pulmonary hypoplasia secondary to congenital kidney disease. All neonates in our study were cannulated onto ECMO within the first 10 days of life. Venoarterial ECMO was utilized for all but one neonate. The average duration of ECMO was 16.6 (±8.9) days. About one half (53.3%) of neonates cannulated onto ECMO did not survive. See Table 1 for additional patient characteristics and ECMO specifications.

**Table 1:** Association of demographic, ECMO, and clinical characteristics with significant cholestasis.

Characteristic	No significant cholestasis (*n* = 24)	Significant cholestasis (*n* = 6)	*p*-value
	*N* (%)	*N* (%)	
Sex			>0.9
Male	17 (70.83)	4 (66.67)	
Female	7 (29.17)	2 (33.33)	
Gestational age in weeks, mean (SD)	37.91 (1.97)	37.50 (1.68)	0.6
Birth weight in grams, mean (SD)	2,905 (590)	2,936 (371)	0.8
Primary disease process			0.2
Congenital diaphragmatic hernia	16 (66.67)	2 (33.33)	
Pulmonary hypoplasia secondary to renal disease	3 (12.50)	3 (50.00)	
Other	5 (20.83)	1 (16.67)	
ECMO type			>0.9
Venous-arterial	23 (95.83)	6 (100.00)	
Venous-venous	1 (4.17)	0 (0.00)	
Oxygenator type			**0.019**
Pediatric quadrox	10 (41.67)	6 (100.00)	
Adult quadrox	14 (58.33)	0 (0.00)	
Pump type			>0.9
Affinity	22 (95.65)	6 (0.00)	
Bio-console	1 (4.35)	0 (0.00)	
Unknown	1 (4.35)	0 (0.00)	
Day of life cannulated onto ECMO, mean (SD)	1.75 (2.77)	1.17 (2.40)	0.5
Venous cannula size (Fr)			0.11
8	3 (12.50)	0 (0.00)	
10	7 (29.17)	4 (66.67)	
12	13 (54.17)	1 (16.67)	
13	1 (4.17)	0 (0.00)	
14	0 (0.00)	1 (16.67)	
Arterial cannula size (Fr)			**0.046**
6	1 (4.35)	0 (0.00)	
8	10 (43.48)	6 (100.00)	
10	12 (52.17)	0 (0.00)	
Days on ECMO, mean (SD)	14.50 (8.10)	24.83 (7.11)	**0.017**
Percent ACT within goal, mean (SD)	0.55 (0.24)	0.70 (0.15)	0.087
Average RPM, mean (SD)	1,650.21 (181.11)	1785.71 (168.42)	0.093
Average ECMO flow in LPM, mean (SD)	0.35 (0.06)	0.34 (0.03)	0.4
Blood Transfusions in mL/kg			
Packed red blood cells, mean (SD)	22.85 (18.65)	36.82 (33.81)	0.3
Platelets, mean (SD)	17.26 (14.10)	17.15 (6.26)	0.5
Plasma, mean (SD)	19.49 (20.33)	17.15 (9.13)	0.5
Survival to discharge	11 (45.83)	3 (50.00)	>0.9

*P*-values for categorical variables are for Fisher’s exact test, *p*-values for continuous variables are for Wilcoxon rank sum test. Data presented as *N* (%) for categorical variables unless otherwise stated as Mean (SD). *P*-values in bold indicate statistical significant, *p* < 0.05.

**Figure 1:**
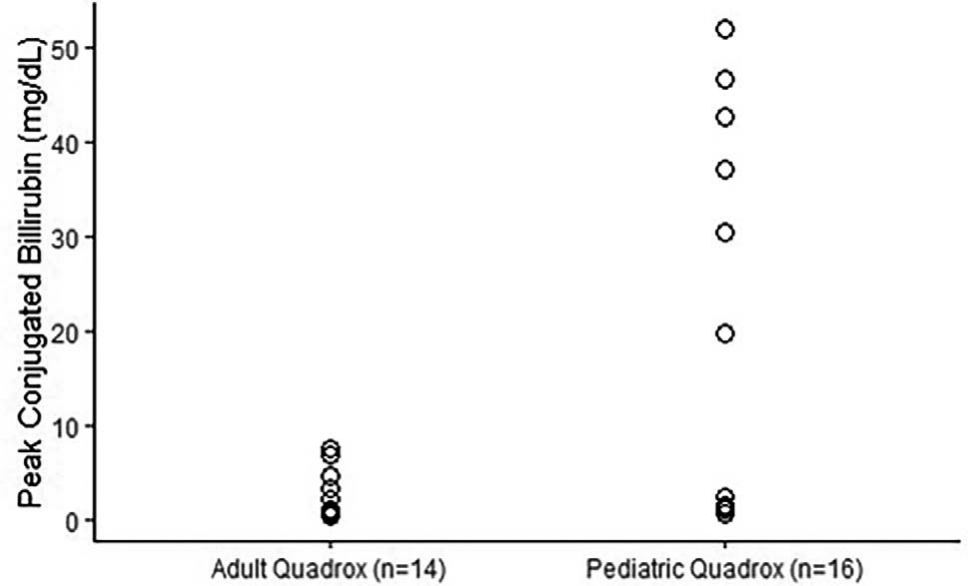
Neonates on pediatric oxygenators had a significantly higher peak conjugated bilirubin (19.7 mg/dL vs. 2.1 mg/dL) compared with those on adult oxygenators.

Sixty percent of the patients in our study developed cholestasis based on the NASPGHAN definition, which is a peak conjugated bilirubin of 1.0 mg/dL or greater. Twenty percent (*N* = 6) of the patients in our study developed severe cholestasis (peak conjugated bilirubin > 10.0 mg/dL). The median conjugated bilirubin level was 1.1 mg/dL with an interquartile range of 5.6 mg/dL (Figure 1).

**Supplementary Figure 1:**
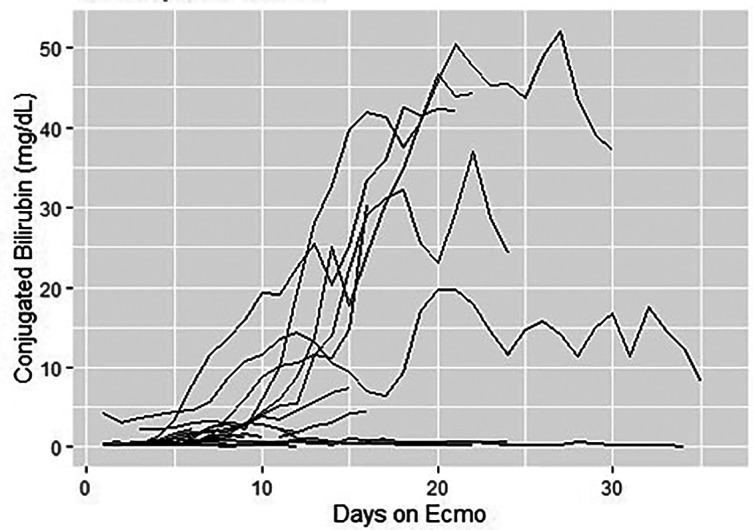
The average correlation between conjugated bilirubin and time on ECMO across all patients was *r* = 0.55.

In a multivariate logistic model using Firth’s correction and with covariates utilizing AIC, we found that pediatric oxygenators and increased duration of ECMO were associated with an increased odds of cholestasis (Table 2). Pediatric oxygenators were associated with a significant increase in odds, with 27.5 times higher odds of significant cholestasis compared with adult oxygenators (*p*-value = 0.01) after controlling for venous cannula size, percentage of time within ACT goal, and days on ECMO. We found that all ECMO cases with severe cholestasis utilized pediatric oxygenators. The median peak conjugated bilirubin for neonates on pediatric oxygenators was 1.4 mg/dL (IQR = [0.9, 32.0]) and the median peak conjugated bilirubin for neonates using adult oxygenators 2.1 mg/dL (median = 0.8, IQR = [0.4, 3.0]), indicating a large difference at the 75th percentile (Figure 1). Furthermore, each additional day on ECMO was associated with a multiplicative increase in the odds of cholestasis of 1.13 (*p*-value = 0.04). In a multivariate linear model, it was found that pediatric oxygenators, increased percentage of time within the ACT goal, and days on ECMO were associated with increased peak conjugated bilirubin (Table 3). Once again, this relationship was large for pediatric oxygenators with the estimated peak conjugated bilirubin begin 14.23 mg/dL higher for pediatric oxygenators than for adult oxygenators (*p*-value = 0.01) after controlling for venous cannula size, percent ACT within goal, and days on ECMO. Each 1% increase in the percentage of time within the ACT goal led to an increase of 0.27 mg/dL in peak conjugated bilirubin. Each additional day on ECMO was associated with an increase in peak conjugated bilirubin of 0.63 mg/dL. The average correlation between conjugated bilirubin and time on ECMO across all patients was *r* = 0.55 (Supplementary Figure 1). Venous cannula size was not significantly associated with cholestasis in either model.

**Table 2:** Estimated odds ratio for cholestasis for logistic model using firth’s correction.

Variable	Odds ratio	Odds ratio	Odds ratio	*p*-value
		CI LB	CI UB	
Oxygenator (Pediatric)	27.51	2.07	4299.18	**0.01**
Venous cannula size	1.47	0.74	4.91	0.29
Percentage of time within ACT goal	1.04	0.99	1.12	0.11
Days on ECMO	1.13	1.01	1.33	**0.04**

CI LB = Confidence interval lower bound, CI UB = Confidence interval upper bound. *P*-values in bold indicate statistical significant, *p* < 0.05.

**Table 3:** Estimated effect on peak conjugated bilirubin for linear model.

	Coefficient	CI LB	CI UB	*p*-value
Oxygenator (Pediatric)	14.23	4.71	23.76	**0.01**
Venous cannula size	2.09	−1.28	5.47	0.21
Percentage of time within ACT goal	0.27	0.05	0.48	**0.02**
Days on ECMO	0.63	0.1	1.17	**0.02**

CI LB = Confidence interval lower bound, CI UB = Confidence interval upper bound. *P*-values in bold indicate statistical significant, *p* < 0.05.

**Supplementary Figure 2:**
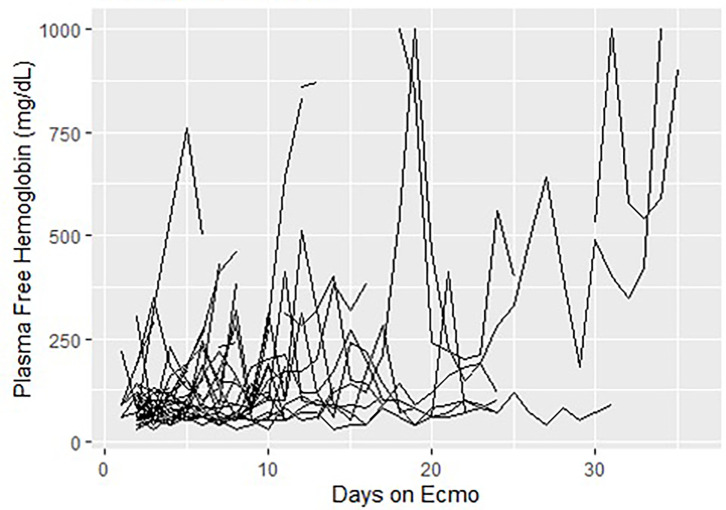
The average correlation between PFH and time on ECMO across all patients is 0.35.

Plasma-free hemoglobin levels were significantly higher in ECMO cases with pediatric oxygenators. D-dimer and fibrinogen levels were not significantly different between cases on pediatric vs. adult oxygenators (Table 4). When we control for plasma-free hemoglobin, the relationship between oxygenator type and conjugated bilirubin level has a lower point estimate (11.6 mg/dL vs. 14.2 mg/dL), indicating that hemolysis may be related to the association between oxygenator type and cholestasis. The average correlation between PFH and time on ECMO across all patients is 0.35 (Supplementary Figure 2).

**Table 4:** Markers of hemolysis by oxygenator type.

	Adult oxygenator	Pediatric oxygenator	*p*-value
	(*n* = 14)	(*n* = 16)	
D-dimer mean (mg/dL)	6.9	10.6	0.08
Plasma free hemoglobin mean (mg/dL)	110.4	204.6	**0.01**
Fibrinogen mean (mg/dL)	318.7	299.5	0.28

*P*-values reported for two-sample *t*-test. *P*-values in bold indicate statistical significant, *p* < 0.05.

An ultrasound of the liver was completed in all neonates with severe cholestasis, and in cases of relative clinical stability, a liver biopsy was completed. There was no clear etiology of cholestasis elucidated from hepatic ultrasound or liver biopsy. Upon decannulation from ECMO, bilirubin levels normalized for all neonates, indicating the lack of intrinsic liver disease as a significant contributing factor.

## Discussion

This is the first study to describe a cohort of neonatal ECMO cases with severe conjugated hyperbilirubinemia. Our analysis identified hemolysis-mediated circuit factors, days on ECMO, and potential effects of anticoagulation as contributing factors.

The strongest contributor to the development of severe cholestasis in our cohort of patients was the use of the Getinge Pediatric Quadrox-iD oxygenator. This pediatric oxygenator was introduced to our institution to reduce the circuit priming volume and surface area of the circuitry and the subsequent risk of inflammation [15, 16]. With these advantages, pediatric oxygenators have become widespread in neonatal and pediatric ECMO.

In reviewing the cases of severe cholestasis in the setting of this specific pediatric oxygenator, we hypothesized that the relationship between pediatric oxygenators and cholestasis was mediated by hemolysis. In a 2015 study by Williams et al., after running an *ex vivo* model of ECMO for 6 h, there were significantly higher levels of plasma-free hemoglobin in the pediatric oxygenator model relative to the adult model [17]. To assess this hypothesis in our study, we compared markers of hemolysis in pediatric and adult oxygenators. We found significantly increased levels of plasma-free hemoglobin in Getinge pediatric oxygenators relative to adult oxygenators (Table 4). Furthermore, when plasma-free hemoglobin is controlled for, the effect of oxygenator type on peak conjugated bilirubin is attenuated. This is consistent with the effect of oxygenator type on bilirubin being partially mediated by hemolysis. There was no statistically significant difference in D-dimer levels between the oxygenator types. This lack of statistical significance may be due to the small sample size limiting the statistical power, as well as the inability of laboratory assays to report D-Dimer values greater than 20. The association between fibrinogen and oxygenator type was not statistically significant. However, fibrinogen is a less specific marker of hemolysis given its multifaceted roles in mediating an inflammatory response [18]. These results suggest that cholestasis in our cohort was partially mediated by hemolysis.

The etiology of increased hemolysis from pediatric oxygenators remains elusive. Two potential hypotheses include hemolysis secondary to increased pressure gradients across the oxygenator and hemolysis from oxygenator-induced shear stress. The data related to these factors is mixed. In the aforementioned *ex vivo* study by Williams et al., there was an increased pressure gradient across pediatric oxygenators relative to adult oxygenators to generate equivalent flow rates that have been found to be significantly associated with increased hemolysis [17, 19]. In contrast, an *in vitro* study by Venema et al., found that there was no statistically significant relationship between transoxygenator pressure drop and hemolysis [20]. Unfortunately, the ECMO system that was used at our institution during the study period did not have the ability to measure pressure gradients, and therefore, these values could not be reported. Another hypothesis for the etiology of oxygenator-induced hemolysis is that turbulent blood flow around the coils and fibers of the oxygenator leads to shear stress with subsequent hemolysis. However, while theoretically plausible, this has not been found to be true in multiple studies [20–22]. Thus, further analyses are needed to assess the etiology of increased hemolysis secondary to pediatric oxygenators.

While there was a significant difference between plasma-free hemoglobin in adult oxygenators and pediatric oxygenators, the plasma-free hemoglobin remained significantly elevated in the adult oxygenator group, reflecting a higher baseline level of hemolysis in the cohort overall. This suggests that there are other etiologies beyond oxygenator type that are contributing to the elevated plasma-free hemoglobin.

We found that the duration of ECMO, anticoagulation status, and cannula size were significantly associated with cholestasis, likely mediated by hemolysis. Another potential circuit-induced factor includes utilization of centrifugal pumps, which has been associated with increased rates of hemolysis [6–8]. We were unable to study this relationship in our study, as all patients in our study, with the exception of one, received ECMO therapy with a centrifugal pump.

Longer durations of ECMO were significantly associated with the development of cholestasis in univariate, multivariate logistic regression, and linear models. This finding is consistent with prior studies [4, 5]. The relationship between longer durations of ECMO and cholestasis is likely multifactorial due to both physiologic and circuit-related factors, as longer ECMO runs are related to both more severe patient physiology and prolonged exposure to the ECMO circuit. Furthermore, percentage of ACT within the goal was associated with the risk of cholestasis in the linear model, although not significant in the multivariate logistic model. This was contrary to what would be expected. We hypothesized that inadequate anticoagulation would lead to clot formation, and therefore increased hemolysis and higher conjugated bilirubin levels. Of note, assessment of anticoagulation status is difficult in the setting of significant conjugated hyperbilirubinemia, as bilirubin interferes with the anti-Xa colorimetric assay. Therefore, the anti-Xa cannot be reported with severe cholestasis and therefore cannot be used to manage heparinization. Because ACT reflects coagulation variables other than heparin effect and is less specific in the neonatal population, variation in anticoagulation management is likely to occur, leading to both potential thrombotic and bleeding complications [23, 24]. Another possibility for this unexpected finding is that the time within the ACT goal was mediated by the longer duration of ECMO. With longer ECMO duration, there is increased time for titration of anticoagulation to be within the ACT goal. This is supported by ACT within goal being statistically significant in the linear model but not the multivariate logistic model when ECMO duration is accounted for. Thus, further research is required to assess the relationship between coagulation status and cholestasis.

Related to the hemolysis evaluation, smaller arterial cannula sizes were associated with increased risk of significant cholestasis in a univariate model, but not in models utilizing AIC for selection. Interestingly, venous cannula size was selected into the model. With the small sample size of our study, it is possible that the arterial cannula size was not chosen for the model due to a correlation between the arterial and venous cannula sizes (*r* = 0.44). In multivariate logistic regression and linear models, venous cannula size was not found to be significantly associated with peak conjugated bilirubin. Thus, in our study, it is unclear whether arterial and venous cannula sizes are associated with hemolysis. There are mixed data on the impact of cannula sizes on hemolysis and subsequently, cholestasis [10, 25, 26].

Lastly, although the primary disease type as an indication for requiring ECMO therapy was not statistically associated with the development of severe cholestasis, it is notable that a greater proportion of neonates with congenital kidney disease developed severe cholestasis (50%) compared to patients with congenital diaphragmatic hernia (33%) or other diseases (17%). Although conjugated bilirubin is primarily excreted via bile through the enterohepatic system, a small proportion is excreted in the urine, especially during states of significant conjugated hyperbilirubinemia [2]. The patients with significant kidney disease and minimal innate kidney function may be more likely to develop and sustain significant peak conjugated bilirubin levels due to a lack of clearance in the presence of hemolysis. Furthermore, critically ill neonates on ECMO with congenital kidney disease require dialysis utilizing continuous renal replacement (CRRT). This is accomplished by attaching a hemofilter to the ECMO circuit. This has been proposed as a risk factor for hemolysis. However, studies have demonstrated inconsistent results on the relationship between CRRT in ECMO and the risk of hemolysis, and subsequently cholestasis [17, 27, 28]. Larger studies including neonates with a variety of indications for ECMO may help to better understand this relationship.

Although previous literature reports an increase in mortality with cholestasis complications on ECMO [7, 8, 29–32], our cohort did not demonstrate an association between severe cholestasis and survival to discharge. Because our data indicate that the severe cholestasis cases are mediated by circuit and anticoagulation factors, particularly the pediatric oxygenator, it makes sense that these patients, once decannulated from the ECMO circuit, may have a similar survival rate as cases without severe cholestasis. Further studies are needed to assess the relationship between severe cholestasis, hemolysis, and mortality.

### Limitations

This study is limited in its retrospective nature and non-overlapping time periods of utilization of pediatric and adult oxygenators at a single institution. With the small sample size, it is challenging to control for all potential confounding variables in the multivariate model. Single-center data reflective of ECMO equipment from a single manufacturer and based on institutional purchasing decisions. For example, although we identified the association of severe cholestasis with the Getinge Pediatric Quadrox-iD oxygenator, that does not necessarily mean that other models of pediatric oxygenators would be associated with similar results. Regarding primary disease type and indication for ECMO, our patient cohort may not be representative of the broader indications for ECMO in the nation. For example, we did not include cardiac indications for ECMO as they were cared for in the cardiovascular ICU and not the NICU, potentially introducing other variables. Also, our study had a disproportionate number of patients with renal pathology due to our institution being the only hospital with pediatric nephrologists in the geographic area.

Anticoagulation strategies are also worth noting. All patients in this cohort received heparin infusions as their primary anticoagulation agent. Studies indicate that the choice of anticoagulant can impact hemolysis [33]. Although the optimal anticoagulant and monitoring targets in the pediatric population are not known [34, 35], newer agents such as Bivalirudin may be effective and have been reported in cases with heparin resistance or severe hemolysis [36–39]. Lastly, there are other physiologic factors, such as sepsis and Multiple Organ Dysfunction Syndrome (MDOS), that may have led to significant cholestasis that was not captured in our study. Furthermore, all but one case utilized the Affinity centrifugal pump and were placed on VA ECMO. Both factors are associated with an increased risk of cholestasis. Future studies with larger sample sizes and analyzing different modes of ECMO are imperative, given the risks of cholestasis on morbidity and mortality.

## Conclusion

Our study described a cohort of neonatal ECMO cases complicated by severe cholestasis that was likely mediated by hemolysis due to circuit factors, particularly the use of the Getinge Pediatric Quadrox-iD oxygenator, as well as longer ECMO duration and anticoagulation management. Further studies are needed to elucidate further other potential contributors, including both patient and circuit factors, so that ECMO therapy can be optimized in this vulnerable neonatal population.

## Data Availability

The research data are available on request from the authors.

## Supplementary material

The supplementary materials of this article are available at https://ject.edpsciences.org/10.1051/ject/2025031/olm.
